# Complement System in Alzheimer’s Disease

**DOI:** 10.3390/ijms222413647

**Published:** 2021-12-20

**Authors:** Akash Shah, Uday Kishore, Abhishek Shastri

**Affiliations:** 1 Barts and The London School of Medicine and Dentistry, Queen Mary University of London, London E1 2AD, UK; a.shah@smd17.qmul.ac.uk; 2 Biosciences, College of Health, Medicine and Life Sciences, Brunel University London, Uxbridge UB8 3PH, UK; 3 Central and North West London NHS Foundation Trust, London NW1 3AX, UK; abhishek.shastri@nhs.net

**Keywords:** neuroinflammation, Alzheimer’s disease, complement system, microglia

## Abstract

Alzheimer’s disease is a type of dementia characterized by problems with short-term memory, cognition, and difficulties with activities of daily living. It is a progressive, neurodegenerative disorder. The complement system is an ancient part of the innate immune system and comprises of more than thirty serum and membrane-bound proteins. This system has three different activating pathways and culminates into the formation of a membrane attack complex that ultimately causes target cell lysis (usually pathogens) The complement system is involved in several important functions in the central nervous system (CNS) that include neurogenesis, synaptic pruning, apoptosis, and neuronal plasticity. Here, we discuss how the complement system is involved in the effective functioning of CNS, while also contributing to chronic neuroinflammation leading to neurodegenerative disorders such as Alzheimer’s disease. We also discuss potential targets in the complement system for stopping its harmful effects via neuroinflammation and provide perspective for the direction of future research in this field.

## 1. Introduction

Alzheimer’s disease (AD) is the most frequent form of dementia in the elderly and accounts for approximately 60–70% of all cases [1,2]. AD is the most prevalent neurodegenerative disorder in the elderly which results in a slow and progressive decline in both memory and executive cognitive functions [2,3]. Some of the core clinical features are progressive memory loss, apraxia (inability to perform movements and gestures), agnosia (inability to recognise and identify people, objects, and sounds), language decline, and behavioural changes such as the loss of executive brain functions [2]. Unfortunately, there are still no treatments available to stop the pathophysiological processes or even progression of AD [2,3].

### 1.1. Epidemiology of Alzheimer’s Disease

According to the World Health Organization (WHO), there are approximately 50 million people worldwide who suffer from dementia; it is estimated that 30 million people have AD [1]. There are approximately 10 million new cases of dementia diagnosed each year across the globe, approximately 6 million cases are of AD [1]. One of the major risk factors for dementia is age; with increasing life expectancy, cases of dementia are only predicted to rise further. There is a higher prevalence in females, probably due to higher life expectancy compared with males [4,5].

### 1.2. Risk factors of Alzheimer’s Disease

AD is a multifactorial disease; it is considered to be caused by an interplay of multiple risk factors such as lifestyle, environmental, and genetic factors [6]. These factors interact with one another to cause the onset or alter the progression of sporadic late onset AD (LOAD), which forms the majority of cases in individuals over the age of 60–65 years [7]. Other types of rare AD cases are known as young onset familial AD, which occurs in individuals before the age of 60, particularly between 30 and 60 years of age [6,7]. These young onset familial AD cases generally have multiple individuals in one generation who have AD; these cases are predominantly linked to mutations in autosomal dominant genes such as the amyloid-β precursor protein (*APP*), Presenilin 1 (*PS1*), or Presenilin 1 (*PS2*) genes [7,8].

Cardiovascular disease can combine with potentially modifiable lifestyle related risk factors and influence the pathogenesis of AD [7,8,9]. A recent study identified 12 modifiable risk factors: hypertension, obesity, diabetes, smoking, depression, physical inactivity, hearing impairment, lack of education, low social contact, excessive alcohol consumption, air pollution, and traumatic brain injury (TBI) [8]. Meta-analyses of these risk factors showed that they may account for approximately 40% of dementia cases across the world [7,8]. Cardiovascular disease can lead to vascular damage of the brain, which increases the risk of macro- and microvascular damage and subsequent brain atrophy [7,8]. Further insults by modifiable risk factors such as diabetes and metabolic syndrome can result in atherosclerosis, brain infarctions, while patients with obesity and insulin-resistance show dysregulation of the complement system associated with chronic low-level inflammation of adipose tissue [7,8,10].

Genetic studies have identified cardiovascular disease associated loci which increase the risk of LOAD [9]. The most prominent gene associated with the strongest impact on LOAD is the apolipoprotein ε4 (*APOE-ε4*) gene which is involved in cholesterol metabolism [9,11]. *APOE-ε4* heterozygotes have three times increased risk of LOAD, and homozygotes have a 15 times higher risk [7]. Genome wide association studies (GWAS) have identified multiple single nucleotide polymorphisms (SNPs) which can increase the risk of AD including the ATP-binding cassette sub-family A member 7 (*ABCA7*) and clusterin (*CLU*) that are involved in lipid transport [9,12]. Other low risk loci identified by GWAS include triggering receptors expressed on myeloid cells 2 (*TREM2*), complement receptor type 1 (*CR1*), bridging integrator 1 (*BIN1*), cathepsin D (*CTSD*), and CD33 [6]. 

### 1.3. The Pathophysiological Changes in Alzheimer’s Disease

AD starts decades before any clinical symptoms manifest [13]. In this preclinical phase, individuals are often clinically asymptomatic but show evidence of AD neuropathology [13]. This preclinical phase can last for decades until later the individual goes through the clinical phases where symptoms of AD begin to manifest and progress [13].

Our current understanding of AD includes two key neuropathological hallmarks—amyloid plaques and neurofibrillary tangles (NFTs) [13]. Amyloid plaques are composed of extracellular deposits of amyloid beta (Aβ) peptides [13]. The Aβ plaques can be diffuse amorphous nonfibrillar Aβ aggregates and/or neuritic plaques. Neuritic plaques are composed of Aβ arranged into β-pleated sheets [13]. Intraneuronal NFTs are composed of aggregates of paired helical filaments (PHFs) of abnormally hyperphosphorylated tau (p-tau) protein [14]. P-tau then aggregates within the neuron leading to the formation of NFTs [14].

It is now clear that neuroinflammation plays a significant role in AD pathophysiology. Studies have shown the presence of reactive glial cells such as microglia and astrocytes, and inflammatory mediators including of the complement system around Aβ plaques. This indicates a significant role for neuroinflammation in AD progression and subsequent neurodegeneration [15,16].

#### 1.3.1. Aβ and the Amyloid Hypotheses

Since the amyloid hypothesis proposed by Hardy and Allsop et al. (1991), Aβ has been thought to be the main causative factor for AD [17], who identified a mutation in the amyloid precursor protein (*APP*) gene on chromosome 21, and suggested that *APP* mis-metabolism and Aβ deposition were key drivers in the pathological cascade of AD [17,18]. The hypothesis suggests that misfolding and deposition of Aβ results in the formation of extracellular Aβ plaques, this leads to tau becoming abnormally hyperphosphorylated becomimg p-tau, leading to the eventual formation of NFTs, and then neuronal death [17]. The hypothesis was further supported by the demonstration of the presence of Aβ in *APP* mutant transgenic mice [19].

**Figure 1:**
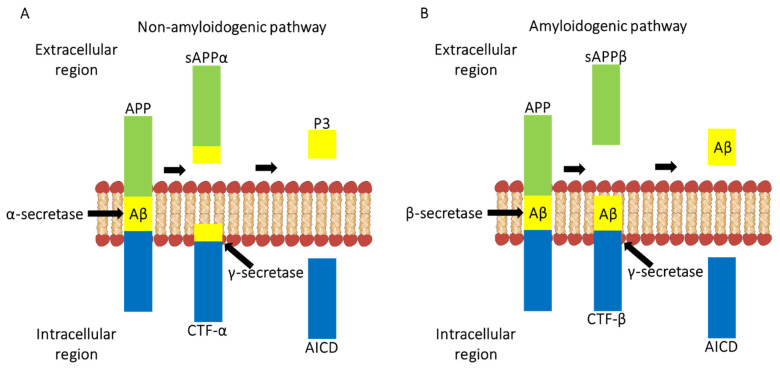
A schematic diagram showing how the amyloid precursor protein can be cleaved via non-amyloidogenic pathway (**A**) and amyloidogenic pathway (**B**). (**A**) In the non-amyloidogenic pathway, *APP* is hydrolysed by α-secretase which produces CTF-α and sAPPα. CTF-α is bound to the membrane, and sAPPα, a soluble ectodomain. CTF-α is then cleaved by γ-secretase within the membrane, allowing the release of P3 and *APP* intracellular domain (AICD). (**B**) In the amyloidogenic pathway, *APP* is hydrolysed by β-secretase 1 producing sAPPβ and CTF-β. CTF-β is then cleaved within the membrane by γ-secretase yielding Aβ peptides- Aβ1-40 or Aβ1-42.

The *APP* is a 695 amino acid-long glycoprotein which can be cleaved by three pathways via α-, β-, γ-, and η-secretases resulting in the production of C-terminal fragments (CTF) [20] (Figure 1). The non-amyloidogenic pathway is where *APP* is hydrolysed by α-secretase which produces products such as CTF-α, which is bound to the cell membrane, and APP-α (sAPPα) which has a soluble ectodomain [20,21]. Both CTF-α and sAPPα are thought to have a neurotrophic and neuroprotective role [20,21]. Following this, CTF-α is cleaved inside the membrane by γ-secretase allowing the release of a peptide, known as P3 [20,21]. P3 is a soluble peptide which does not tend to aggregate unlike Aβ [21]. The non-amyloidogenic pathway does not produce Aβ peptides [18,20] (Figure 1).

In the amyloidogenic pathway, *APP* is hydrolysed by β-secretase 1 (BACE1) and produces CTF-β which is bound to the cell membrane [18,20,21] (Figure 1). CTF-β is then cleaved within the membrane by γ-secretase yielding Aβ1-40 or Aβ1-42 peptides which are released extracellularly [22]. The variation in length of the peptides is dependent on which site γ-secretase cleaves CTF-β [22]. The Aβ1-42 peptide is the most soluble neurotoxic peptide and most prone to aggregate and subsequently forms Aβ plaques [15,21]. The third pathway allows *APP* hydrolyses via η-secretase and is thought to be an alternative pathway of processing under physiological conditions [18,20].

Normally in a healthy individual, the *APP* is predominantly hydrolysed via the non-amyloidogenic pathway [18,21]. However, Aβ peptides can be produced via the amyloidogenic pathway to serve some important functions such as the regulation of synapses and post-TBI recovery [18]. The majority of Aβ peptides which are produced are mostly the Aβ1-40 isoform, but Aβ1-42 peptides are also present [15]. Any excess Aβ production is cleared by activating glial cells and the complement system via phagocytosis [18].

Failure to clear Aβ1-42 peptides by the immune system due to the chronic neuroinflammation results in impaired microglial mediated Aβ clearance. This leads to an increase in concentration and aggregation of Aβ1-42 peptides which causes neurotoxic effects such as the release of proinflammatory cytokines and complement proteins, leading to microglia-mediated neuronal death and synaptic destruction [18]. In AD, metal ion homeostasis is disrupted which results in elevated levels of trace metal ions such as copper [23]. Copper is also seen within Aβ plaques [23]. Interestingly, in a recent study, a correlation was observed between plasma Cu^2+^ levels and complement components such as C3 and C4 (see below for further details on complement systems and neuroinflammation). Copper is a metal ion with redox properties and the copper (Cu^2+^) ions within Aβ plaques can produce hydrogen peroxide and reactive oxygen species (ROS) via biological reducing agents such as ascorbic acid, dopamine, and cholesterol [23,24]. ROS can cause dendritic and axonal atrophy leading to neuronal dysfunction; this is of importance in key areas of the brain such as the hippocampus [18,25,26]. The neurotoxic Aβ1-42 peptides are thought to be the key steps in the pathological drive leading to AD [18,25]. It is thought that aggregation of Aβ peptides begins in vivo decades before any clinical symptoms manifest [18,25]. These peptides act on several receptors such as *N*-methyl-D-aspartate (NMDA) and α7-nicotinic acetylcholine (α7-nACh) which causes the inhibition of long-term potentiation (LTP) in brain regions such as the hippocampus and enhances long-term depression (LTD) [27]. Inhibition of LTP can result in the shrinkage of dendritic spines and their gradual loss [25]. This leads to the dysfunction of synapses and has a direct impact on learning and memory [18,25]. Aβ1-42 peptides also induce hyperphosphorylation of tau, further supporting the idea that that Aβ can drive tau pathology [13,28].

#### 1.3.2. Tau, Neurofibrillary Tangles and the Tau Propagation Hypothesis

The tau protein is normally found in neuronal axons in the brain and belongs to a family of microtubule-associated proteins [18]. The main function of tau is to stabilise microtubules that allows axonal transport, maintenance of synaptic structures and function, and signaling between neurons [18]. Tau is produced by the alternative splicing of exons 2, 3 and 10 of the Microtubule-Associated Protein Tau (*MAPT*) gene which can produce six tau isoforms [18,29]. These isoforms contain several microtubule binding carboxyl terminals with repeats of either three or four arginine residues, which is to prevent tau from aggregating [18,29]. Tau is known to be a phosphoprotein which can be phosphorylated and dephosphorylated by protein kinases and phosphatases, respectively [18,29]. Tau has a few phosphorylation sites. It has been shown that Aβ1-42 can activate glycogen synthase kinase 3β (GSK3β), also formerly known as Tau protein kinase 1 (TPK1), which leads to the abnormal hyperphosphorylation of tau and formation of p-tau, which can polymerise and leads to the formation of PHFs and eventually NFTs; these processes further exacerbate AD pathology [28,30]. NFTs are neurotoxic as they disrupt microtubule function and cause subsequent impairment in axonal transport leading to synapse and neuronal damage and eventual neurodegeneration [13].

The tau propagation hypothesis was first proposed in 2009 by Frost et al. [31], which suggests that the progression of cognitive impairment in AD is linked to the spreading of tau pathology in the brain [18]. It is thought that p-tau can more easily propagate in a prion-like manner from one area of the brain to other areas; thus, spreading tau pathology in the brain which leads to the activation of glial cells, neuroinflammation and neurodegeneration [18,31]. This was supported by animal studies where brain extracts from P301s tau transgenic mice were injected into the brains of ALZ17 mice that only normally develop tau pathology late in life; it was found that these mice developed tau pathology much quicker than their respective wildtype (WT) mice [18,32].

It is established now that Aβ is a strong driver for AD pathology. However, Aβ alone may not be enough to impact on cognition but it can also drive the downstream pathology such as the eventual formation of p-tau and formation of NFTs which can affect cognition [13].

#### 1.3.3. Role of Neuroinflammation in AD

Neuroinflammation can be defined as an inflammatory response within the central nervous system (CNS) in response to infection, trauma, and even neurodegenerative diseases [16]. Neuroinflammation is mediated by the activation of glial cells such as astrocytes and microglia, and the production of cytokines, chemokines, ROSs, and the activation of the complement system [16]. Some pro-inflammatory cytokines include IL-1β, IL-6, and tumour necrosis factor alpha (TNFα), and some chemokines include C-C motif chemokine ligand 2 (CCL2), CCL5, and CXCL1 (C-X-C motif chemokine ligand 1) [16]. These inflammatory mediators are produced by the innate immune cells of the brain- astrocytes and microglia [16]. However, it is important to note that these inflammatory mediators can also be recruited from other parts of the body if there is a disruption in the blood-brain-barrier (BBB) due to trauma or ageing resulting in the loss of its mechanical integrity at the later stages of life [16].

The release of the pro-inflammatory mediators by innate immune cells can result in the dysfunction of synapses, inhibition of neurogenesis and neuronal death; these features are also seen in AD [16,33,34]. Anti-inflammatory cytokines such as IL-4, IL-9, IL-10, IL-11, and transforming growth factor (TGF)-β1 are also produced during neuroinflammatory events to potentially maintain a homeostatic balance to prevent excessive neuroinflammation [16,35,36]. It is now evident that inflammatory mechanisms play a role in the pathophysiology of AD [16,35,36]. It is understood that the effects of neuroinflammation are neuroprotective only when it is low-level or present for a short time [3]. Neuroprotective benefits include immune surveillance by glial cells, thereby protecting from infections, synaptic pruning which helps in improving cell-to-cell transmission, the remyelination process, and the production of neutrophin that helps in neuronal growth [37]. However, chronic neuroinflammation can occur when the neuroprotective mechanisms become overwhelmed by Aβ and NFTs that make microglia and astrocytes more hyperactive which can exacerbate AD pathology [37].

The deposition of Aβ is a key neuropathological hallmark and is a key initiating event in the pathophysiology of AD [13]. The formation of Aβ plaques in the hippocampus is of key importance as it is involved in short-term memory processing; the recruitment of neurotoxic Aβ peptides results in disruption in homeostasis, synaptic dysfunction and astrocyte and microglia hyperactivation [13,15,16]. Further insults by Aβ and a failure of clearance can result in an increase in Aβ1-42 peptides which also have the ability to bind to α-amino-3-hydroxy-5-methyl-4-isoxazolepropionic acid (AMPA) receptors and calcium ion (Ca^2+^) channels, leading to an increase in intracellular Ca^2+^ which over time leads to chronic neuroinflammation and production of ROS and complement proteins via microglia [13,15,16].

## 2. Role of the Complement System in CNS

The complement system is an essential component of the innate immune system and acts as a bridge between innate and adaptive immunity [38]. It was first discovered by Jules Bordet in 1896 and described as a heat-labile component of serum which “complemented” antibodies [39]. The complement system comprises of over 40 proteins including control proteins and cell surface receptors all of which are integral to the innate immune system, allowing it to rapidly recognise and clear pathogens, and maintain homeostasis by clearing apoptotic and necrotic cells and debris [16,40].

The complement proteins are used in a hierarchy of sequential proteolytic cascades which are activated when a foreign pathogen, non-self ligand or altered host cells are recognised [39]. The complement can induce an inflammatory response by pro-inflammatory mediators (anaphylatoxins) and “tag” pathogens through a process known as opsonization for phagocytosis by antigen presenting cells (APCs), and target pathogens for lysis via the formation of the membrane attack complex (MAC) [39].

The complement system also has a role in the CNS, which was once thought to be an immune privileged system due to the BBB. Some of the roles of the complement system in the CNS include synapse elimination during early development, synaptic plasticity throughout life, cell migration, and the removal of misfolded proteins [3,41,42,43]. However, when the complement system in the CNS becomes dysregulated, it can be a contributor to neurological, neurodegenerative, and psychiatric diseases [16]. Studies have shown that complement proteins, complement control proteins, and receptors are upregulated in immunohistopathological analysis of post-mortem human brain tissue and cerebrospinal fluid (CSF) [3,16].

### The Complement System

**Figure 2:**
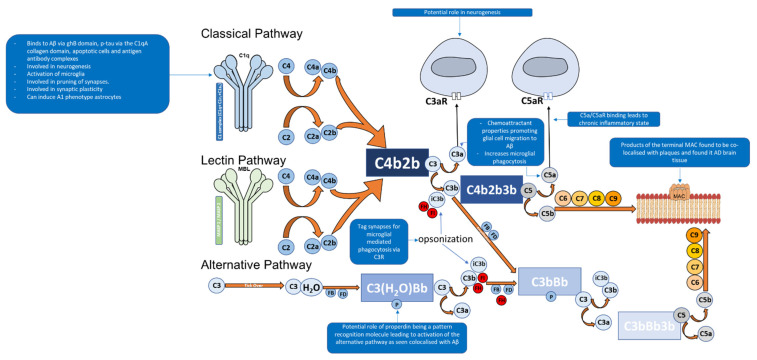
The complement system consists of more than 40 soluble or membrane-bound proteins, and is activated via classical, lectin or alternative pathway. The classical pathway is activated by Aβ andp-tau. Apoptotic cells and antigen-antibody complexes interact with C1q (complexed with C1r_2_ and C1s_2_). C1s then cleaves C4 and C2 and ultimately leading to the formation of C3 convertase C4b2b. The lectin pathway is activated by mannan-binding lectin (MBL) and ficolin, which then recruits MASPs (MBL-associated serine proteases) to cleave C2 and C4, yielding C3 convertase, C4b2b. The alternative pathway is an amplification loop and is also initiated by hydrolysis of C3 to C3(H_2_O) which leads to the formation of C3 convertase, C3bBb. All three pathways lead to formation of C5 convertase (classical and lectin: C4b2b3b; alternative: C3bBb3b) generating C5b which binds to C6, C7, C8, and C9 to form a cell lysing membrane attack complex (MAC). Some of the mechanisms of complement activation in AD are shown in the blue boxes.

Depending on the target ligand, the complement system can be activated by three pathways: classical, alternative, and lectin [15,16,44]. All three pathways share the common central component of the complement system C3, and downstream of C3 results in the formation of the MAC [15] (Figure 2). However, the pathways differ according to the ligand they can bind to [15,44].

The classical pathway of the complement system is activated when the C1 complex (C1q, C1r_2_, and C1s_2_) binds via C1q, the first subcomponent of the classical pathway, to a target ligand such as the Fc region of complement fixing IgG and IgM [15,40,45]. Furthermore, C1q can bind to aggregated Aβ as well as hyperphosphorylated tau [46,47,48] (Table 1).

The C1 complex is composed of C1q (Figure 2), a charge pattern recognition molecule, and a tetramer of serine proteases C1r_2_ and C1s_2_ [15,40,44,45]. C1q serves as a molecular scaffold to C1r_2_ and C1s_2_; binding of C1q to a target ligand autoactivates C1r, which subsequently cleaves and activates C1s [44]. The activated C1s then goes on to cleave C4 to generate C4a and C4b which are an anaphylatoxin and opsonin, respectively [40,49]. C4b then recruits C2 [40]. C1s then cleaves C2 in to C2a and C2b [40]. The cleaved products C4b and C2b form C4b2b, a proconvertase complex [40,49,50]. The proconvertase complex is cleaved by C1s which results in C3 convertase, C4b2a [40,51]. C3 convertase cleaves multiple C3 proteins in to C3a and C3b [38,40]. C3b serves two functions, the first being an opsonin where it will covalently attach to the surface of a pathogens and drives the amplification of the complement alternative pathway, which results in opsonisation and phagocytosis of the tagged pathogen [38,40].The second function is where C3b leads to the downstream activation of the terminal pathway forming the MAC [38,40]. This is performed by C3b associating with C4b2b which results in the formation of C4b2b3b, known as C5 convertase of the classical pathway [40]. C5 convertase then cleaves C5 into C5a (an anaphylatoxin that also acts as a chemoattractant) and C5b, the latter facilitates the formation of the C5b, C6, C7, C8, and C9 complex (C5b-9), known as the MAC [37,38,40,52]. The MAC can then bind to the cell membrane and cause cell lysis [15].

The alternative pathway of the complement system is an essential amplification loop and the pathway is regulated by several control proteins, factors B, D, H, I, and properdin [53] (Figure 2). C3b molecules generated from any of the three pathways can covalently attach to the cell surface of a pathogen. C3b associates with factor B which results in the formation of C3bB, a Mg^2+^ dependent proconvertase complex [53]. Factor B is then cleaved by factor D to form Bb and Ba [54]. This results in the formation of C3bBb, which is the C3 convertase of the alternative pathway [53,54]. C3bBb cleaves C3 to C3a, an anaphylatoxin (which can also act as a chemoattractant) and C3b, of which C3b forms the alternative pathway amplification loop forming a new C3bBb complex each time [53,54].

Furthermore, the alternative pathway is continuously initiated by the slow hydrolysis of C3 to C3(H_2_O) [53]. This is achieved by the hydrolysis of the internal thioester bond within C3 by water in plasma [53]. This is known as the “tick over theory” which was proposed by Lachmann et al. [53,55]. C3(H_2_O) has also been described as “C3b like” molecule by Pagburn et al. [53,56]. The hydrolysis of C3 to C3(H_2_O) causes a structural change which allows the binding of Factor B (FB) [56]. This C3(H_2_O) and FB-bound complex is cleaved by a protease, Factor D (FD), resulting in the formation of Ba and Bb [54]. The larger Bb fragment remains associated with the C3(H_2_O) complex resulting in the formation of C3(H_2_O)Bb, a C3 convertase protease complex [54]. This complex can cleave additional C3 molecules through its serine protease domain which results in the formation of C3a and C3b [54]. The C3b molecule can function as an opsonin or bind to C3 convertase (C3bBb), resulting in the formation of C5 convertase (C3bBb3b) of the alternative pathway, followed by formation of the MAC [54].

Both types of C3 convertases generated by the alternative pathway have a very short half-life, and therefore, require stabilisation by a binding partner called properdin [57] (Figure 2). When properdin binds to C3bBb, it can increase its stability/half-life by up to 10-fold; but this is less for C3(H_2_O)Bb [57,58]. Additionally, properdin may be able to act as a pattern recognition molecule which can bind to microorganisms and initiate the alternative pathway [59,60]. This can potentially explain properdin colocalising with Aβ plaques and other complement proteins in an AD mouse model, and more importantly, C1q^−/−^ mice AD [61].

The alternative pathway can be regulated by control proteins such as Factor H (FH) and factor I (FI) [15,16,62]. FH is an essential soluble regulator of the alternative pathway where it competes with FB for binding to C3b, thus prevents the formation of C3 convertase (C3bBb) by promoting the disassociation of Bb from C3bBb. FH is also known as the decay accelerating factor of the alternative pathway which can downregulate activity of the alternative pathway [63]. The stabilisation of the alternative pathway C3 convertases though properdin distorts the binding site of FH [15,16,62,64]. FH also has the ability to act as a cofactor for FI which results in the irreversible degradation of C3b to iC3b (inactivated C3b) which is not able to bind to FB [15,16,62,64]. FH is composed of 20 complement control proteins (CCPs): CCPs 1–4 allow the functional activity of FH which include decay acceleration by disassociating Bb from C3bBb, cofactor activity for FI and binding to C3b in an extended configuration [65]. Additionally, the FH affinity is increased through glycosaminoglycans and sialic acid, both expressed on self-cells, which may explain how FH can discriminate between self and nonself cells and prevent self-damage by the complement system [65]. The affinity of FH for C3b increases via CCP sites 1–4, 7–15 and 19–20; SNPs within these sites are considered to contribute to neuroinflammation and a potential role in AD pathophysiology [15,16,65].

The lectin pathway of the complement system is activated through pattern recognition molecules such as mannane-binding lectin (MBL) and ficolins, which bind to oligosaccharides on the surface of pathogens [66,67,68]. These pattern recognition molecules have an N-terminal collagenous region similar to C1q, but the C-terminal region differs as they contain C-type lectin domains [66,67,68]. Once activated, associated enzymes mannan-binding lectin serine protease 1 (MASP1) activates MASP2 which goes on to cleave C2 and C4 in to C2a, C2b, C4a and C4b; of which C2a and C4b form C3 convertase, C4b2a [66,67,68]. This pathway can then lead to the eventual formation of the MAC.

**Table 1:** Functions of complement system in the central nervous system.

Functions of Complement System	Role	Mechanism	Reference
Neuroprotection	Neurogenesis	Increased complement receptor activation in the development of cerebellar neurons in animal models.	[67]
		Disrupting C3aR signalling in mice models impairs neurogenesis.	[68]
		CR2 is a negative regulator of neurogenesis.	[67,68]
	Synaptic pruning	C1q^−/−^ mice exhibit increased synaptic connections resulting in epilepsy, indicating an essential role in synaptic pruning.	[69,70]
	Synaptic plasticity	C1q^−/−^ mice show weak dendrites and spines.	[69]
Neuroinflammation	Binding with Aβ	Activation of classical pathway.	[42,43,71]
	Binding with Tau protein	Activation of complement system via classical pathway.	[44,72]
	Interaction with microglia	Neuronal death due to release of proinflammatory cytokines.	[15,29,30,73]
		C1q released by microglia can induce A1 astrocytes.	[73]
		Presence of complement receptors can increase phagocytosis.	[74,75,76]
	Interaction with astrocytes	Neuronal death due to release of pro-inflammatory cytokines.	[73,77]
		Neurotoxic A1 astrocytes can activate the classical pathway and release pro-inflammatory cytokines.	[73,78,79,80]
	NF-κB pathway activation via Aβ	Increased release of C3 via activation of NF-κB pathway resulting in microglia activation and release of pro-inflammatory cytokines.	[81,82]

## 3. Complement System and Alzheimer’s Disease

### 3.1. Role of the Complement System in Central Nervous System Physiology

The CNS was thought to be an immune privileged system due to the BBB [16]. However, it is now known that complement components can be produced within the CNS by astrocytes, microglia, and neurons [16,69,70]. The complement system can be neuroprotective as well as neurotoxic, dependent on initiating targets and the level of activation [16] (Table 1).

The complement system has been demonstrated to have a role in neurogenesis. This was indicated in an in vivo study by Bénard et al., who identified an increased expression of C3aR and C5aR in 12-day old rat cerebellar neurons, suggesting these receptors were involved in neurogenesis [16,83] (Table 1). This was supported by Raphpeymai et al. who demonstrated an impairment of neurogenesis in C3^−/−^ and C3aR^−/−^ mice, suggesting an involvement of C3aR signalling in neurogenesis [16,71]. Complement receptor 2 (CR2) also has a role in neurogenesis in adult neural progenitor cells; CR2^−/−^ mice show increased hippocampal neurogenesis, thus CR2 seems to be a negative regulator of hippocampal neurogenesis [16,71,83] (Table 1).

C1q has been shown to play a role in synaptic pruning and synaptic plasticity [42]. C1q^−/−^ mice show an increase in synaptic connections; increased synaptic connections in C1q^−/−^ mice can result in epilepsy [42,72,84]. C1q^−/−^ mice also show increased synaptic plasticity in the regions of the brain such as the hippocampus, which are associated with neurodegenerative diseases such as dementias [42,72,84] (Table 1).

### 3.2. The Specific Role of the Complement System in Alzheimer’s Disease

The very important role of the complement system in AD pathophysiology is supported by neuropathology observed in vitro and in vivo. Studies have revealed that complement protein expression and complement activation lead to neuroinflammation, neuronal and synapse loss and subsequent neurodegeneration which is seen in AD patients. Complement proteins have been colocalised with Aβ plaques. A post-mortem study conducted by Rogers et al. (1992), who analysed AD patients brain tissues, showed elevated levels of C1q, C3, and C4 co-localisation with Aβ plaques compared with control samples [47] (Table 1). Another study observed elevated levels of C3 and C4 mRNA in the temporal cortex of AD brains [85]. A study by McGeer et al. (1989) identified an abundance of positive immunohistochemical staining of C1q, C3, and C4 and their colocalization with Aβ plaques and NFTs in AD brain tissue [52]. Specific staining for complement activation products such as C3b and the products of the terminal MAC (C5b-C9) in AD brain tissues has also been reported, indicating that that MAC can potentially cause neuronal loss and neurodegeneration in AD [85].

It is likely that the complement dysfunction may be contributing to neuroinflammation and subsequent neurodegeneration decades before clinical symptoms manifest in an individual with AD; this can be due to Aβ accumulation which overwhelms the complement system and drives the pathology of AD. In vitro studies have demonstrated that Aβ1-42 can directly activate the classical pathway by binding to C1q via its globular domain [86]. C1q can also bind to tau via the C1qA collagen domain and activate the classical pathway [87]. Thus, complement activation due to the binding of C1q to Aβ and tau can potentially contribute to neuroinflammation and neurodegeneration in AD.

Animal models have allowed reproduction of the hallmarks of AD. Studies on C3 gene-deficient mice (C3^−/−^) that had a nerve injury showed a faster recovery compared with WT mice, indicating the complement system is involved in synapse removal and hinders recovery [88]. Another study examined the role of the complement on synapses in ageing mice and observed that C3^−/−^ mice had better learning and memory in regions of the brain such as the hippocampus compared with their respective aged WT mice, suggesting that C3 or downstream complement components play a role in hampering synapses as a part of the aging process [89]. However, other studies have suggested that the complement system is essential for synaptic pruning [72,84] (Table 1). The complement system in a normal brain may aid in synaptic plasticity throughout life, but in the later stages of life, insults and accumulation of Aβ and NFTs can overactivate the complement or cause its dysfunction and fail to clear the hallmarks of AD ^3^.

An in vivo study used PS1/APP mouse model, a cross between transgenic mice carrying a mutation in the *APP* (APP_K670N,M671L_) which exhibits increased levels of Aβ deposition in the hippocampus and cerebral cortex, and mutant PS1 transgenic mice which have no pathological changes but show mildly elevated levels of Aβ1-42 and Aβ1-43 peptides [90,91,92]. The resulting PS1/APP transgenic mice showed an accelerated Aβ accumulation and deposition providing a useful amyloid phenotype of AD [92,93]. The study reported that C1q co-localised with Aβ plaques and activated microglia [92]. This was supported by another study which showed that C1q bound to Aβ and caused phagocytosis via microglia [94]. The role of C1q was examined further by Fonseca et al. (2004), who crossed AD mice model Tg2576 mice (*APP* mutation) with C1q^−/−^ mice, giving rise to APPQ^−/−^ mice which exhibited AD pathology but lacked C1q, and these mice were compared with APP mice [90,93,95,96]. At older ages, both types of mice developed Aβ. However, activated forms of microglia were significantly lower in APPQ^−/−^ mice compared with Tg2576 mice which indicated that C1q can have a potential detrimental effect on neurons by activating both microglia and the classical pathway [95].

Hong et al. (2016) found that human APP (hAPP) (J20) transgenic mice, a mouse model which overexpresses hAPP with mutations linked to familial AD (Swedish and Indiana mutations) yielding vast amount of Aβ plaques at young age, showed an increase in C1q from approximately 1 month of age compared with its WT mice, and this was prior to the formation of Aβ plaques. This posed the question if C1q increase is dependent on soluble Aβ levels [97,98,99]. When the J20 mice were administered “compound E”, a γ-secretase inhibitor which rapidly decreased Aβ levels, there was a marked reduction in C1q levels, implying that C1q levels and activation of the complement can be dependent on Aβ levels [97]. When WT mice were injected with Aβ oligomers, there was a loss of synaptic density within 72 h [97]. However, this was not the case in C1q^−/−^ mice which indicated that the inhibition (absence) of the classical pathway led to reduced neurotoxic effects of Aβ [97]. Thus, C1q-mediated classical pathway activation is increased by Aβ peptides that triggers the downstream pathway leading to neurodegeneration [97]. A recent study examined the relationship between synapse dysfunction and synapse loss in conjunction with C1q [100]. The study used APP/PS1 mice and control WT mice, both types of mice had fluorescent C1q tags [100]. The study observed a decline in mitochondrial function and changes in the septin protein structure which aids in synaptic transmission in the area where C1q was tagged, suggesting that the synapse loss was mediated by the complement system [100].

Shi et al. (2017) examined the role the complement on synapses and cognition in C3^−/−^ AD mice (APP/PS1-C3 KO) [101]. The mice were obtained by breeding C3^−/−^ mice with APP/PS1 mice [101,102,103]. The APP/PS1- C3^−/−^ mice had an abundance of Aβ in late-stage AD and they performed significantly better in cognitive tasks compared with APP/PS1 mice [101]. Additionally, there was a marked reduction in pro-inflammatory cytokines such as IL-12, TNFα and Interferon gamma (IFN-γ), and decreased activation of microglia (as evident from microglial immunoreactive markers CD68 and Iba-1) despite the abundance of Aβ, suggesting that early complement components in the presence of Aβ can mediate the downstream activation of the complement system which can result in gliosis, neuroinflammation and neurodegeneration [101].

## 4. Role of Glial Cells in AD and the Complement System

Astrocytes constitute approximately 30% of the cells in the CNS; they can morphologically be found in two forms, protoplasmic (in grey matter) and fibrous (in white matter) [16,104]. Astrocytes contribute to the BBB via their astrocytic end-feet, which line the surface of the brain and form a covering around the cerebral vessels and synapses [105,106]. Astrocytes also have a role in neuronal development and synaptogenesis, providing metabolic support to synapses [104,105]. Insults to the CNS causes astrocytes to change their morphology to become reactive astrocytes which exhibit hypertrophy of their processes and upregulate the release of glial fibrillary acidic protein (GFAP) and S100B; all of which are seen in AD brain tissue analysis [16,77]. Studies on AD human brains have identified reactive astrocytes in close proximity to Aβ plaques, and astrocytes containing Aβ plaques have also been stained positive; this may indicate a potential role of astrocytes in the clearance of Aβ in the early stages of AD [107,108]. Animal model studies have demonstrated that astrocytes migrate to Aβ via chemokines such as monocyte chemoattractant protein-1 (MCP-1), present in Aβ plaques and that astrocytes bind to and degrade Aβ [73]. Injection of Aβ oligomers induced a strong activation of astrocytes via the nuclear factor-kappa B (NF-κB) leading to the production of pro-inflammatory cytokines IL-1β and TNFα, surrounding injection sites and in close proximity to blood vessels which can draw in further glial cells, contributing to neuroinflammation [78] (Table 1). Furthermore, astrocytes have the ability to phagocytose Aβ [79,80]. Astrocytes exist in two reactivity states: A1 and A2 [81]. A2 astrocytes are neuroprotective as they perform homeostatic functions that helps restore activity of neurons and synapses after insults, whereas A1 astrocytes fail to perform this and convert to a neurotoxic form [81,82]. These neurotoxic A1 astrocytes can significantly increase activation of the complement classical pathway [81] (Table 1). Additionally, pro-inflammatory cytokines such as IL-1 and TNFα, and complement components including C1q, which are released by microglia, can also induce A1 astrocyte phenotype [81]. Extracellular tau aggregates can bind to astrocytes, get internalised via an integrin α_V_/β_1_ receptor; the integrin signalling pathway causes NF-κB activation leading to the release of several pro-inflammatory cytokines and chemokines that converts astrocytes to an A1-like neurotoxic state [82,109,110]. The NF-κB pathway is also implicated by another study where exposure to Aβ activates this pathway and causes an increased release of C3 which can bind to neuronal and microglia C3aR, resulting in microglia activation, and release of proinflammatory cytokines [111,112]. C3 transcripts are also known to be upregulated in A1 astrocytes [81] (Table 1).

Post-mortem examination of AD brain tissues revealed an abundance of A1 astrocytes which also stained positive for C3 in brain regions affected by AD [81]. Furthermore, C1q was found to induce the A1 phenotype of astrocytes in vivo [81]. Following traumatic nerve crush injuries, C1q^−/−^ mice showed a significant decrease in A1 astrocytes in comparison with WT controls [81], suggesting C1q influence on A1 astrocyte phenotype

**Figure 3:**
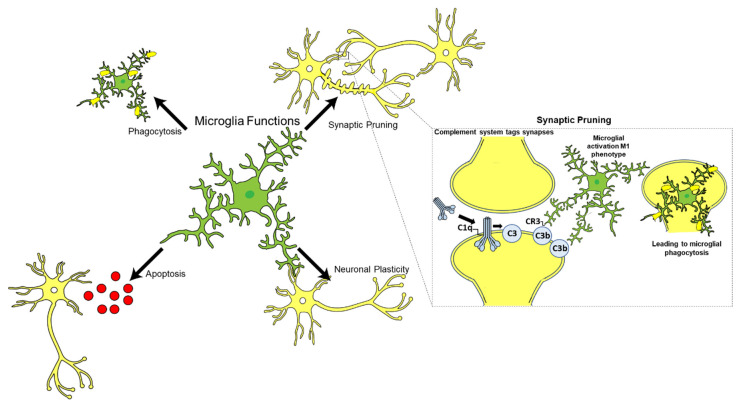
Microglial cells (shown in green) under physiological conditions have ramified appearance and are the resident immune cells of the central nervous system (neuronal cells shown in yellow). Microglia are involved in a variety of functions such as pruning of synapses, phagocytosis of pathogens, apoptotic and necrotic cells, and regulation of neurogenesis and neuronal plasticity, thus helping in overall effective functioning of the central nervous system. During development of the brain, synaptic pruning takes place where the complement system aids microglia in the removal of weak synapses. However, in AD, neurons and glial cells synthesise and secrete complement proteins such as C1q. C1q can bind to synapses and cause activation of the complement classical pathway leading to cleavage of C3 to yield C3b, an opsonin which will tag synapses. Microglia recognise the tagged C3b neurons via their CR3 receptor and phagocytose the neurons which can eventually lead to neurodegeneration. (Figure adapted from Dalakas, M.C.; Alexopoulos, H., Spaeth, P.J. Complement in neurological disorders and emerging complement-targeted therapeutics. Nat Rev Neurol. 2020, 16, 601–617).

Microglia are the resident immune cells of the CNS and constitute approximately 10–15% of the total glial cell population in the adult human brain (Figure 3) [113]. Microglia play an essential role in immune surveillance in the CNS; they have long-ramified processes which they use to survey the microenvironment for cellular debris, pathogens, and misfolded proteins, and provide tropic support to the brain [114,115]. Other functions of microglia include neurogenesis, apoptosis, synaptic plasticity, and synaptic pruning in conjunction with the complement system, particularly C1q (Figure 3) [116,117].

It is generally accepted that microglia can exist in two predominant phenotypes upon activation: M1 and M2 [118]. The classical M1 phenotype is associated with an upregulation of pro-inflammatory cytokines such as IL-1, IL-6, TNFα and C1q which can result in a neurotoxic environment as they can activate A1 astrocytes, forming a vicious pro-inflammatory positive feedback loop [81,104,118]. This also correlates with studies involving TBI and subsequent neuroinflammation [119]. The alternative M2 phenotype is associated with secretion of anti-inflammatory cytokines such as IL-4, IL-9, IL-10 and transforming growth factor beta (TGF-β) [81,104,118] (Table 1).

Microglia can perform immune surveillance in the brains via several innate immune pattern recognition receptors (PRRs) including scavenger receptors (such as CD36), Toll-like receptors (TLRs), receptors for advanced glycosylation end products (RAGE) and other receptors such as CD14 and CD47 [120,121,122]. PRRs can responds to insults via damage associated molecular patterns (DAMPs) and pathogen associated molecular patterns (PAMPs), which have the ability to recognise cellular debris, pathogens and misfolded proteins such as Aβ and induce a microglial response [16].

Aβ is also able to bind TLR-2, 4, 6, and 9 resulting in microglial activation and the subsequent release of pro-inflammatory cytokines and chemokines which recruit more microglia to the site [123,124]. Antibody blocking of TLR-2 results in reduced pro-inflammatory cytokine production [124]. In TLR-2^−/−^ mice, Aβ peptides were not able to induce a pro-inflammatory response [124].

Balducci et al. examined the role of Aβ on TLR-4 and its effects on microglial activation in mice [125]. When mice were provided a cerebroventricular injection of Aβ peptides, this activated microglia and a marked increase in pro-inflammatory cytokines such as TNFα [125]. The study compared the effects of Aβ on TLR-4^−/−^ mice and control WT mice who were injected with Aβ [125]. Aβ had a devastating impact on memory in WT mice, while no changes in memory were found in TLR-4^−/−^ mice, implicating TLR-4 in microglial activation and neuroinflammation [125]. In another study, TLR-4 and TLR-6 were implicated along with CD36, in the inductionof microglia in the presence of Aβ [123]. A study Scholtzova et al. (2017) used a TLR-9 agonist: class B cytosine–phosphate–guanine (CpG) oligodeoxynucleotides (ODNs) in a triple transgenic mice model (Tg-SwD), which had significant cerebral amyloid angiopathy (CAA) and observed that CpG ODN reduced CAA and improved cognition, implicating TLR-9 in microglial activation [126]. Studies have also demonstrated that Aβ can activate microglia via the CD36, CD14, and CD47 receptors [120]. The release of certain types of pro-inflammatory cytokines such as TNFα upon Aβ binding can result in an increase in phagocytic activity of microglia which can further exacerbate neuroinflammatory events by activating A1 astrocytes and via C1q which can bind to Aβ plaques, resulting in synapse elimination and eventual neurodegeneration [74].

Chemokines, and anaphylatoxins such as C3a and C5a, which have chemoattractant properties, are also implicated in AD. One study examined the chemokine receptor CX3CR1 which is expressed on microglia and allows Fractalkine (CX3CL1) binding which recruits more microglia to sites of neuroinflammation, e.g., Aβ plaques in AD [127]. Heterozygous PS1/APP-CX3CR1^+/−^ mice, compared with age-matched PS1/APP mice, had a significant reduction in Aβ plaques in the brain compared with the control mice, and levels of Aβ degrading enzymes such as insulin-degrading enzyme and matrix metalloproteinase 9 which are produced by neurons, were also significantly reduced, suggesting a potential therapeutic target to promote Aβ clearance [127].

Several in vivo and in vitro studies have been conducted to determine how complement proteins are produced in the brain. Early in vitro studies of glial cell lines suggested the possibility of the synthesis and secretion of complement proteins [128,129]. The presence of complement receptors on microglia for C1q, C3a, and C5a, was also noted which could enhance microglial phagocytosis [130] (Table 1). The complement system and microglia can exist in harmony in the absence of insults in a neuroprotective manner. CR3^−/−^ mice have a defect in microglia-mediated synapse elimination compared with controls. This highlighted the important role of microglia and the complement in normal brain development [75]. However, in AD, the activation of the complement system and microglia can be detrimental to neurons. This was demonstrated in an in vivo study where Aβ was added to glial neuronal cultures, resulting in CR3 mediated neuronal loss. However, this was prevented by blocking CD11b, a component of CR3, possibly showing a therapeutic target for CR3 [76].

C1q has also been shown to be detrimental to neurons in the presence of Aβ. Microglia are the main source of C1q in the adult brain and its levels increase with age [131]. C1q can bind to Aβ and trigger the classical pathway; C1q can also bind to neuronal insult sites, resulting in local apoptosis and pro-inflammatory cytokine release [132]. In response to this, microglia can take on the M1 phenotype and become activated and express receptors for C3a and C5a which further increases local inflammation. The sites which are C1q tagged can be opsonised by C3b and then phagocytosed by the M1 microglia; this has been demonstrated in animal model studies [42]. Some recent studies have determined a role of tau in microglial activation. In a tau mouse model upon administration of a C1q antibody, microglia induced synapse loss was prevented and synaptic density was recovered [133,134]. Additionally, the deletion/absence of C3aR on microglia led to the reduction in neuroinflammation and thus neurodegeneration [133,134].

## 5. Therapeutics

Several clinical trials have taken place and are underway to find a cure for AD (Table 2). Some of the most promising trials for AD were based on the Aβ cascade hypothesis. Aβ targeting monoclonal antibodies (mAbs) such as solanezumab progressed to phase III clinical trials [18,135]. Solanezumab was able to bind to soluble Aβ and this was achieved in AD patients where plasma concentrations of Aβ decreased by approximately 90% but cognition still deteriorated [18,135]. Other mAbs such as gantenerumab and crenezumab succeeded in targeting Aβ but failed to prevent cognitive decline [18,136]. Aducanumab is another mAb which was shown to target neurotoxic Aβ peptides and progressed to phase III studies, named EMERGE and ENGAGE [137]. The EMERGE study used higher doses compared with the ENGAGE study and thus showed a mark reduction in cognitive decline, reduction in Aβ plaques and NFTs identified from positron emission tomography (PET) scans and p-tau reduction was identified from CSF analysis [137]. The ENGAGE study only yielded positive results such as the EMERGE study, in individuals treated with a higher dose for a longer period of time [137]. However, despite positive results, the trials were stopped by the sponsor due to efficacy related issues between both studies [137].

A more promising approach may be to target neurotoxic Aβ peptides which have been post-translationally modified with a pyroglutamate at the N-terminus (pGlu3, pE3) [138]. Hettmann et al. generated an anti-pGlu3-Aβ antibody, called PBD-C06, which was shown in vitro to strongly bind to pGlu3-Aβ peptides [138]. The advantage of this antibody is that it will not react with *APP* nor with other non-pathological Aβ peptides in the brain or periphery, thus it has minimal toxic effects [138]. Furthermore, the peptide is designed by making a K322A substitution on the Fc region which essentially keeps the Fc binding integrity but inhibits the C1q binding; this can prevent activation of the complement system and thus prevent a microglial response in individuals with AD [138]. It has been difficult to identify tau targets as they are an essential component of microtubules in the CNS. However, studies are being undertaken to identify drugs which can fall into these categories: tau kinase inhibitors, tau aggregation inhibitors, microtubule stabilises and immunotherapies [18]. Tau aggregation inhibitors such as TRx0237 are currently in phase III clinical trials and so far in 9 months of treatment in AD patients, general atrophy of the brain slowed down compared with control patients [18,139] (Table 2).

From the information above, it is known that TNFα, a proinflammatory cytokine, can result in the formation of M1 microglia and release of C1q. Adalimumab is a mAb against TNFα and has been studied via in vivo animal models [140]. Park et al. (2019) examined if adalimumab can improve cognition and reduce AD pathology in an Aβ injected mouse model of AD [140]. The Aβ injected mice were treated with the mAb and significant improvement in memory was noticed in comparison with Aβ alone injected control mice [140]. In the Aβ injected mice being treated with the mAb, there was also a reduction in BACE1 as well as Aβ plaques [140]. Adalimumab is a biological drug which is licensed to be used in many inflammatory conditions such as rheumatoid arthritis, psoriatic arthritis, ankylosing spondylitis, Crohn’s disease and ulcerative colitis [2]. Thus, it can be possible to conduct a prospective study on individuals who are receiving adalimumab if any of them go on to develop AD. It can also be worthwhile investigating if AD patients with above-mentioned inflammatory diseases who are being treated with adalimumab show improvement in their cognition

Complement inhibition can potentially help to slow down the progression of AD. Complement inhibition was investigated in a study by Lee et al. (2017), who used PMX205 as a C5aR antagonist in hSOD1^G93A^ mice which are a model of amyotrophic lateral sclerosis (ALS) [141]. The study recognized that orally administered PMX205 managed to enter the CNS in a pharmacologically active concentration and improved grip strength in those mice thus slowing the progression of the disease as well as increasing survival. [141].

Inhibition of C1q in animal models has shown an improvement in cognition and reduction in AD pathology, as mentioned above. Many have pondered if an anti-C1q drugs can be a therapeutic for AD or other CNS conditions. However, there is a potential of increased susceptibility to infection and immune complex deposition in various organs following C1q deficiency [2]. Furthermore, high doses would also have to be given to penetrate the BBB to ensure a pharmacologically active concentration [142].

## 6. Conclusions and Future Perspectives

Current evidence on the pathophysiology of AD indicates that the complement system plays both a neuroprotective and neuroinflammatory role. During the early stages of AD when clinical symptoms have not manifested, the complement system can succeed in clearing Aβ in conjunction with glial cells. However, when Aβ starts to accumulate and Aβ plaques develop, the consequent chronic inflammation is detrimental. Glial cells acquire their neurotoxic phenotypes and release C1q which can directly bind to Aβ at synapses and cause a release of pro-inflammatory cytokines. Some of these cytokines have the ability to further increase the phagocytic capability of microglia. Microglia can then engulf the synapses which results in early synaptic loss and then gradual neurodegeneration as seen in AD. This pro-inflammatory cascade can function as a positive loop and lead to more neuroinflammation and neurodegeneration.

Complement proteins and regulators must be further explored. Lee et al. (2017) were successful in using a C5aR antagonist which entered the CNS in a pharmacologically active concentration and slowed the progression of ALS in mice [141]. C1q can pose as a prime target to disrupt the complement system and prevent neuroinflammation and neurodegeneration. Another complement target can be C3aR as it is a key receptor expressed on both neurons and microglia. This would essentially aid in the reduction in the phagocytic activity of microglia and potentially reduce the release of pro-inflammatory cytokines. Another point of the complement system which can be investigated is C3 convertase of the classical pathway, C4b2a. Such regulation of the pathway can prevent the cleavage of C3 to C3b; thereby, preventing the downstream pathway of the complement system and stopping cell lysis and reducing synaptic loss and neurodegeneration.

Furthermore, it may be worthwhile looking into potential brain complement specific biomarkers which can indicate if an individual has an increased susceptibility to develop AD in the future. Additionally, it is worth investigating if any individuals who has an inflammatory condition and have AD benefits in terms of cognition following biological mAb treatment. Clinical trials (Table 2) using mAb targeting Aβ have not showed much desired results in terms of improving cognition or slowing down the progression of AD. One exception is Aducanumab which was recently approved by the FDA for use in individuals with mild AD and a high Aβ plaque load. It may also be worthwhile exploring other potential therapeutic targets for AD, such as p-tau and complement targeted therapies. Promising results have been seen in the trial of TRx0237, a tau aggregation inhibitor which opens a door to potentially exploring other tau targets such as p-tau epitopes. Complement targeted therapies are unlikely to reverse AD, unless picked up very early on, but they can potentially modify or slow the progression of the disease by protecting the brain from chronic neuroinflammation before symptoms begin to manifest. Complement targeted therapies will most likely face the same challenges as mAb such as penetrating the BBB without causing any complications in other systems such as circulatory, respiratory, etc. More research needs to be carried out on mechanisms in prevention or regulation of microglia in acquiring the neurotoxic M1 phenotype by potentially blocking C3aR and C5aR. There is a potential to explore the relationship of C1q and Aβ.

## Figures and Tables

**Table 2:** Summary of the clinical trials for treating AD.

Target	Drug	Additional Trial Information	Reference
Aβ	Solanezumab	Solanezumab, a human monoclonal antibody directed against soluble Aβ. A double blind, placebo-controlled phase 3 trial in individuals with mild AD which was defined by a mini mental state examination score of 20-26 and Aβ confirmation via a positron emission tomography (PET) scan or Aβ1-42 CSF analysis. Approximate 90% reduction in soluble Aβ but cognition continued to decline.	[18,132]
	Gantenerumab	Gantenerumab, a human monoclonal antibody directed against Aβ aggregates. A double blind, placebo-controlled phase 3 trial in individuals with AD selected via several neuropsychological analysis, MRI, CSF analysis and PET scan. Study halted early due to futility. Higher doses of gantenerumab may be needed to clinical efficacy.	[18,133]
	Crenezumab	Crenezumab is a monoclonal antibody which can bind to Aβ fibrils, monomers and oligomers. Phase 3 trials terminated early as cognition continued to decline.	[18]
	Aducanumab	Aducanumab, a monoclonal antibody that targets Aβ. Study showed high affinity for neurotoxic Aβ. Phase 3 trials named EMERGE and ENGAGE. EMERGE study identified a reduction in cognitive decline, Aβ plaques, NFTs and p-tau. Recently approved by U.S. Food and Drug Administration (FDA) for patients with early AD and Aβ plaque build.	[134]
Tau	TRx0237	TRx0237, a low dose leuco-methylthioninium bis(hydromethanesulphonate) (LMTM) is a tau aggregation inhibitor. Currently in phase 3 trials in patients with mild AD. So far results have shown a reduction in general brain atrophy compared with control patients.	[136]

## Data Availability

Not applicable.
